# Serotonin receptors and suicide, major depression, alcohol use disorder and reported early life adversity

**DOI:** 10.1038/s41398-018-0309-1

**Published:** 2018-12-14

**Authors:** Mark D. Underwood, Suham A. Kassir, Mihran J. Bakalian, Hanga Galfalvy, Andrew J. Dwork, J. John Mann, Victoria Arango

**Affiliations:** 1 Department of Psychiatry, Columbia University College of Physicians and Surgeons, New York, NY USA; 2 Division of Molecular Imaging and Neuropathology, Columbia University and New York State Psychiatric Institute, New York, NY USA; 3 Division of Biostatistics, Columbia University and New York State Psychiatric Institute, New York, NY USA; 4 Department of Pathology and Cell Biology, Columbia University College of Physicians and Surgeons, New York, NY USA; 5 Macedonian Academy of Sciences and Arts, Skopje, Macedonia

## Abstract

Serotonin neurotransmitter deficits are reported in suicide, major depressive disorder (MDD) and alcohol use disorder (AUD). To compare pathophysiology in these disorders, we mapped brain serotonin transporter (SERT), 5-HT_1A_, and 5-HT_2A_ receptor binding throughout prefrontal cortex and in anterior cingulate cortex postmortem. Cases and controls died suddenly minimizing agonal effects and had a postmortem interval ≤24 h to avoid compromised brain integrity. Neuropathology and toxicology confirmed absence of neuropathology and psychotropic medications. For most subjects (167 of 232), a DSM-IV Axis I diagnosis was made by psychological autopsy. Autoradiography was performed in right hemisphere coronal sections at a pre-genual level. Linear model analyses included sex and age with group and Brodmann area as interaction terms. SERT binding was lower in suicides (*p* = 0.004) independent of sex (females < males, *p* < 0.0001), however, the lower SERT binding was dependent on MDD diagnosis (*p* = 0.014). Higher SERT binding was associated with diagnosis of alcoholism (*p* = 0.012). 5-HT_1A_ binding was greater in suicides (*p* < 0.001), independent of MDD (*p* = 0.168). Alcoholism was associated with higher 5-HT_1A_ binding (*p* < 0.001) but only in suicides (*p* < 0.001). 5-HT_2A_ binding was greater in suicides (*p* < 0.001) only when including MDD (*p* = 0.117) and alcoholism (*p* = 0.148) in the model. Reported childhood adversity was associated with higher SERT and 5-HT_1A_ binding (*p* = 0.004) in nonsuicides and higher 5-HT_2A_ binding (*p* < 0.001). Low SERT and more 5-HT_1A_ and 5-HT_2A_ binding in the neocortex in depressed suicides is dependent on Axis I diagnosis and reported childhood adversity. Findings in alcoholism differed from those in depression and suicide indicating a distinct serotonin system pathophysiology.

## Introduction

Impaired serotonin (5-HT) neurotransmission is detectable in the brain of suicide decedents and in the cerebrospinal (CSF) fluid of nonfatal suicide attempters^1,2^, major depressive disorder (MDD) and alcohol use disorder (AUD)^3,4^. Biological findings in suicide include those related to comorbid diagnoses and those associated with the diathesis for suicide that explains why only a subgroup of people with MDD or AUD are at elevated risk for suicide^5,6^. Despite the fact that suicide-associated conditions such as MDD and AUD are frequently co-morbid, little work has compared 5-HT receptor abnormalities in suicide, MDD, and AUD (see ref. ^1^ for review).

Childhood adversity increases the risk of suicide, MDD, and AUD in adulthood^7,8^. What mediates the effect of childhood adversity on the risk for these diagnoses is not well understood, but epigenetic and gene-environment interactions are reported^9–11^. We hypothesize that childhood adversity affects the serotonin system and contributes to increased risk for suicide, MDD or AUD in adulthood.

In vivo and in vitro imaging of the SERT and 5-HT receptor subtype binding in suicide and nonfatal suicide suggest there are serotonin system abnormalities; but there is less agreement about the direction of the findings, the specificity of the findings for each of these conditions, including which brain areas are involved^12–22^. Explanations for discrepant results include small effect sizes, variability in outcome measures, and biodemographic variability related to sex and age; other factors are the heterogeneity in suicide behavior and the effects from comorbid psychiatric disorders. Abnormalities in the serotonin system are more pronounced with more lethal suicidal behavior^16,22–25^. In the present study, we sought to determine the effects of suicide on serotonin receptor binding and separate the effects of suicide from comorbid MDD, AUD, and early life adversity studying postmortem brain using quantitative autoradiography, in the hitherto largest published sample of postmortem suicides and controls.

## Materials and methods

### Subjects

The Division of Molecular Imaging and Neuropathology at the New York State Psychiatric Institute was the source of the brain samples. The Institutional Review Boards of the appropriate Institutions approved all procedures. Subjects had postmortem intervals (PMI) of 24 h or less and died suddenly. The Coroner or Medical Examiner diagnosed suicides on the basis of evidence of intent and a self-inflicted fatal act. There were 232 cases total (Table 1): suicide decedents (*n* = 83) and nonsuicides (*n* = 149). The majority of the cases and controls used have been published elsewhere in smaller, focused studies^4,26–31^.

**Table 1:** All Subject demographics

Group (*n*)	Age (y) Mean ± SEM	Sex (M:F)	Race W:B:A:H	PMI (h) Mean ± SEM	Brain pH Mean ± SEM	Axis I Dx (*n*)	Cause of death (*n*)
Nonsuicides (*n* = 149)	44 ± 1	119:30	98:31:4:16	14.2 ± 0.4	6.54 ± 0.02	None (48) AUD (35/18)* MDD (6) Unknown (43) Other (5)	Respiratory failure (4), Liver failure (3), Cancer (1), Cardiac (71), hemorrhage (4), Gunshot wound (8), Stabbing (8), MVA (34), Fall from height (6), Industrial accident (2), Other accident (2), Drowning (1) Electrocution (1), Other (Natural/Accidental) (2), Asphyxiation (2)
Suicides (*n* = 83)	45 ± 2	62:21	60:8:2:13	15.8 ± 0.7	6.37 ± 0.18	None (3) AUD (14/6)* MDD (45) Unknown (22) Schizophrenia (4)	Fall from height (13), Hanging (29), Gunshot wound (28), Subway (2), Drowning (4), Overdose (2), Asphyxiation (2), Poison ingestion (2), Other (1)

*AUD* alcohol use disorder (dependent/abuser), *MVA* motor vehicle accident; *Some AUD subjects were comorbid for MDD

The next-of-kin of 167 subjects (Table 2) agreed to a psychological autopsy interview^32^. We fully implemented the psychological autopsy protocol only after the first 65 suicides and nonsuicides had been collected. Diagnoses were based on DSM-IV criteria and used the Structured Clinical Interview for DSM (SCID-I and SCID-II^33^), and the Brown-Goodwin Aggression History Scale^34^. AUD diagnosis was based on the psychological autopsy and autopsy findings such as liver cirrhosis combined with a blood or brain alcohol level of >0.15%. Medication use within three months of death were recorded and recent use was confirmed by toxicology. All diagnoses, or lack thereof for controls, were made at a consensus conference with experienced psychiatrists, psychologists and other researchers. In the 232 cases, 65 subjects did not have a psychological autopsy, but we were able to review charts and other medical examiner records and arrive at a consensus diagnosis.

**Table 2:** Subjects with psychological autopsy

Group (*n*)	Age (y) Mean ± SEM	Sex (M:F)	Race W:B:A:H	PMI (h) Mean ± SEM	Brain pH Mean ± SEM	Axis I Dx (*n*)	Cause of death (*n*)
Nonsuicides (*n* = 106)	45 ± 2	91:15	68:22:3:13	14.6 ± 0.5	6.55 ± 0.03	None (48) AUD (35/18)* MDD (6) Other (5)	Respiratory failure (2), Liver failure (2), Cancer (1), Cardiac (59), Hemorrhage (4), Gunshot wound (2), Stabbing (6), MVA (19), Fall from height (3), Industrial accident (2), Drowning (1) Electrocution (1), Other (natural/accidental) (2) Asphyxiation (2)
Suicides (*n* = 61)	45 ± 3	42:17	45:4:1:9	16.6 ± 0.8	6.31 ± 0.3	None (3) AUD (14/5)* MDD (45) Other (4)	Fall from height (8), Hanging (20), Gunshot wound (21), Drowning (4), Overdose (2), Asphyxiation (1), Poison (2), Other (1)

*AUD* alcohol use disorder (dependent/abuser), *MVA* motor vehicle accident; *Some AUD subjects were comorbid for MDD

All cases were sudden-death cases

### Brain collection

After brain removal, the brainstem was separated by a transverse cut at the rostral margin of the superior colliculus. The cerebellum was removed by severing the peduncles. The brain was bisected and the right hemicerebrum was cut into 2cm-thick coronal slabs. The slabs were placed on a glass plate, frozen in liquid Freon 12 (DuPont), placed in labeled plastic bags and transferred to a −80 °C freezer. Cerebellar tissue was collected for genetics and brain toxicology. The left hemisphere was placed in formalin for neuropathology.

### Receptor autoradiography

**Fig. 1:**
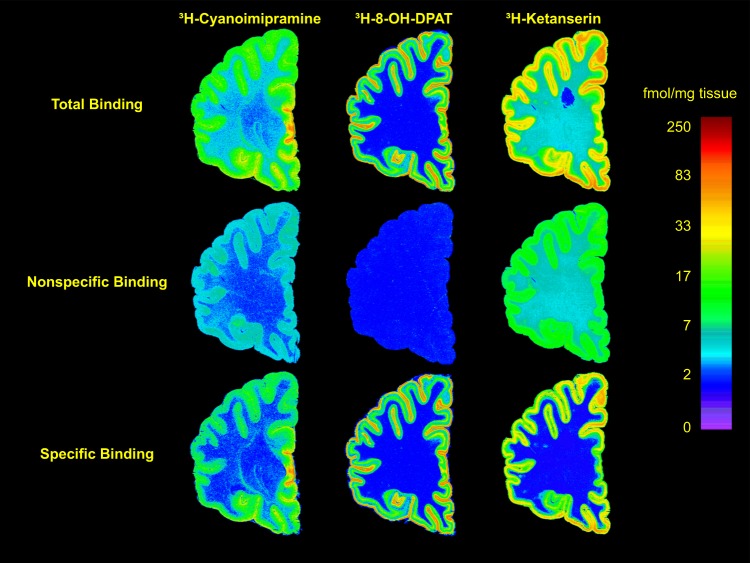
Representative autoradiograms of receptor binding of [^3^H]-Cyanoimipramine to the serotonin transporter (left), [^3^H]-8-OH-DPAT to the 5-HT_1A_ receptor (center) and [^3^H]-Ketanserin to the 5-HT_2A_ receptor (right). The autoradiograms are from sections cut from the right hemisphere of a representative nonpsychiatric control. The upper row has images of total binding, the middle row is of nonspecific binding and the lower row has subtracted images of specific binding. See methods for displacers and assay conditions. The images were calibrated to fmol/mg tissue using radioactivity standards and color-mapped to a single scale on the right

Quantitative in vitro receptor autoradiography was done on frozen tissue sections as described elsewhere^26,27^. A single concentration of ligand was used which was based on the K_d_ reported in the literature and verified previously in our laboratories^26,28^. In brief, 20 µm sections were used for [^3^H]Cyanoimipramine, [^3^H]8-OH-DPAT and [^3^H]Ketanserin binding, to label SERT sites, 5-HT_1A_ and 5-HT_2A_ receptors, respectively (Fig. 1). Six tissue sections were used for each assay, three for total binding and three adjacent sections for non-specific binding. Sections were preincubated in buffer to remove endogenous ligands and incubated with radioligand under optimal conditions. Nonspecific binding was determined by incubation with appropriate displacers. Sections were then washed in buffer (4 °C), dipped in water, rapidly dried and transferred to a vacuum desiccator until exposure (24 h).

Dried slides were exposed to tritium-sensitive film (Hyperfilm from Amersham, or MS film from Kodak). Each film was exposed with tritium standards (American Radiolabeled Chemicals, Inc.). Films were developed (Kodak D-19) for 4 min at 17 °C, rinsed briefly, and fixed (Kodak Rapid Fixer) for 5 min The sections were fixed in buffered formalin and stained with thionin or cresyl violet.

Autoradiograms were quantified using an image analysis system (MCID, Imaging Research, Inc.). Images of standards were calibrated to femtomoles of radioligand per milligram of tissue. Samples of receptor binding were averaged from three sections to produce one binding measure for that individual.

- **[**^**3**^**H]Cyanoimipramine (CN-IMI) binding to serotonin transporter sites:** Total SERT binding was determined with 0.4 nM ^3^H-CN-IMI and nonspecific binding using 10 μM sertraline^26^.
- **[**^**3**^**H]8-OH-DPAT binding to 5-HT**_**1A**_ **receptors:** 5-HT_1A_ receptors were measured using our modifications of the protocol of Hoyer et al.^35^. Slides were incubated with 2 nM [^3^H]8-OH-DPAT and 100 nM sertraline (to block SERT sites).
- **[**^**3**^**H]-Ketanserin (Ket) binding to 5-HT**_**2A**_ **receptors:** Total binding was determined by incubation with 2 nM ^3^H-Ket, 1 μM prazosin and 1 μM tetrabenazine.

### Statistical analyses

Statistical tests were done using SPSS (Version 24, IBM Analytics, NY) and R (Version 3.3.2, R Foundation for Statistical Computing; https://cran.r-project.org).

There were three primary hypotheses: (1) SERT are reduced in suicides; (2) there are more 5-HT_1A_ receptors in the PFC in suicide; and, (3) there are more 5-HT_2A_ receptors in the PFC in suicides. Linear models were used since the response variables were continuous (scalar) (SPSS Procedures UNIANOVA, REGRESSION, and T-TEST). Post hoc tests were performed only when main factors had a significant interaction with brain region. Suicide and MDD were fixed factors, brain regions were assigned as a random factor. All models tested included age and sex as covariates, and when found to be significant, correlation analysis was performed. Statistical tests were performed on raw values. The three receptors (SERT, 5-HT_1A_ and 5-HT_2A_) were examined individually, and while uncorrected *p*-values are reported, a Bonferroni–adjusted significance level of 0.017 was used to preserve an experiment-wise Type I error rate of 0.05 for the primary analyses.

We sought to determine whether any differences in suicide, MDD, AUD or reported early life adversity (ELA) were widespread in PFC or anatomically discrete. The Brodmann areas present at this anatomical level included BA8, BA9, BA46, BA45, BA47, BA11, BA12, BA24, and BA32.

The primary hypotheses were re-tested in a sensitivity analysis limited to subjects with psychological autopsy: Two secondary analyses for each outcome tested whether the difference of binding in suicides is accounted for by the MDD or AUD; a third analysis tested whether binding is associated with aggression or reported early childhood adversity. These hypotheses were made a priori, but being secondary, no *p*-value adjustment was made for multiple testing other than using the 0.017 significance level to adjust for the three outcome measures.

## Results

### Serotonin transporter (SERT)

In nonsuicides (*n* = 143), SERT binding ranged from 5.9 ± 2.1 fmol/mg tissue in BA8 to 23.9 ± 12.0 fmol/mg tissue in BA24 (mean ± SD, Table 3).

**Table 3:** Transporter, 5-HT_1A_ and 5-HT_2A_ binding in suicide and major depressive disorder

All cases were sudden-death cases.											
Receptor	n	Group	BA 8	BA 9	BA 46	BA 45	BA 47	BA 11	BA 12	BA 32	BA 24
SERT	143	NonSuicide	5.86 ± 2.13	6.38 ± 2.6	6.83 ± 2.84	8.81 ± 4.42	9.62 ± 5.22	10.13 ± 5.55	11.21 ± 5.36	9.9 ± 4.29	23.91 ± 11.98
	77	Suicide	5.35 ± 2.61	5.76 ± 2.96	5.81 ± 3.46*	7.65 ± 4.31	8.8 ± 5.43	9.52 ± 6.15*	9.34 ± 5.5*	9.05 ± 6.53	20.49 ± 16.63
5-HT_1A_	143	NonSuicide	13.93 ± 4.77	14.66 ± 4.99	14.24 ± 4.79	14.75 ± 4.64	17.72 ± 5.57	19.18 ± 6.54	18.42 ± 6.2	16.15 ± 5.55	19.45 ± 7.18
	79	Suicide	14.19 ± 4.03	14.59 ± 4.64	14.97 ± 4.61*	15.19 ± 3.86	18.07 ± 4.87	19.68 ± 5.97	18.62 ± 5.43	16.26 ± 3.97	18.49 ± 6.08
5-HT_2A_	145	NonSuicide	29.38 ± 10.82	29.58 ± 10.82	29.36 ± 9.96	30.36 ± 10.55	32.76 ± 11.35	33.13 ± 11.55	33.22 ± 11.52	30.03 ± 10.05	25.48 ± 9.57
	78	Suicide	30.05 ± 12.09	30.34 ± 12.54	31.41 ± 12.59	30.24 ± 11.48	33.65 ± 12.52	33.43 ± 12.55	32.93 ± 12.5	30 ± 11.69	23.21 ± 9.05

Only cases with psychological autopsy:											
Receptor	n	Group	BA 8	BA 9	BA 46	BA 45	BA 47	BA 11	BA 12	BA 32	BA 24
SERT	102	NonSuicide	5.7 ± 2.1	6.3 ± 2.58	6.73 ± 2.73	8.46 ± 4.53	9 ± 5.06	9.55 ± 4.79	10.92 ± 4.95	9.58 ± 4.17	22.54 ± 11.58
	55	Suicide	4.58 ± 2.19	5.33 ± 2.64	5.4 ± 3.07	6.8 ± 3.64	8.11 ± 5.42	8.9 ± 6.5	8.15 ± 4.76	7.61 ± 4.55	15.63 ± 12.95
5-HT_1A_	100	NonSuicide	12.79 ± 3.76	14.01 ± 4.34	13.28 ± 3.84	14.06 ± 4.28	16.51 ± 5.32	17.98 ± 5.85	17.42 ± 5.95	14.81 ± 5.04	18.14 ± 6.43
	56	Suicide	14.68 ± 4.06*	14.88 ± 4.4	15.53 ± 4.34*	15.26 ± 3.55	18.27 ± 4.85*	20.06 ± 5.8*	18.8 ± 4.86	16.09 ± 4.16	19.67 ± 5.96
5-HT_2A_	104	NonSuicide	27.37 ± 9.08	28.42 ± 10.37	29.14 ± 9.88	29.65 ± 10.12	32.1 ± 10.57	32.99 ± 11.3	32.96 ± 11.22	29.35 ± 9.34	24.6 ± 8.74
	56	Suicide	31.09 ± 13.85*	31.67 ± 13.68*	32.82 ± 13.26*	31.76 ± 12.19	35.41 ± 13.15*	35.43 ± 13.03	34.31 ± 13.42	31.07 ± 12.92	23.43 ± 9.82
SERT	112	NoMDD	5.65 ± 2.11	6.33 ± 2.62	6.65 ± 2.71	8.41 ± 4.49	9.11 ± 5.27	9.77 ± 5.24	10.85 ± 4.97	9.63 ± 4.21	21.83 ± 12.17
	47	MDD	4.55 ± 2.17	5.1 ± 2.45*	5.28 ± 3.14*	6.6 ± 3.44*	7.68 ± 4.81	8.2 ± 5.71	7.72 ± 4.52*	7.04 ± 4.27*	16.28 ± 11.94
5-HT_1A_	110	NoMDD	13 ± 3.63	14.14 ± 4.22	13.68 ± 3.93	14.31 ± 4.09	16.92 ± 5.03	18.54 ± 5.6	17.67 ± 5.81	15.04 ± 4.79	18.19 ± 6.07
	48	MDD	14.72 ± 4.49*	14.94 ± 4.7	15.15 ± 4.47*	14.99 ± 3.93	17.71 ± 5.48	19.16 ± 6.52	18.61 ± 4.99	15.96 ± 4.67	19.77 ± 6.77*
5-HT_2A_	112	NoMDD	28.03 ± 10.31	29.02 ± 11.12	29.7 ± 10.63	29.97 ± 10.4	32.79 ± 11.24	33.53 ± 11.67	33.28 ± 11.84	29.63 ± 9.83	24.91 ± 9.03
	50	MDD	29.72 ± 12.27	30.8 ± 12.85	32.22 ± 12.57	31.35 ± 11.78	34.18 ± 12.23	34.19 ± 12.66	33.55 ± 12.34	30.61 ± 12.31	21.6 ± 8.39

Values are expressed as fmol/mg tissue, mean ± SD

*p < 0.05

#### Suicide

**Fig. 2:**
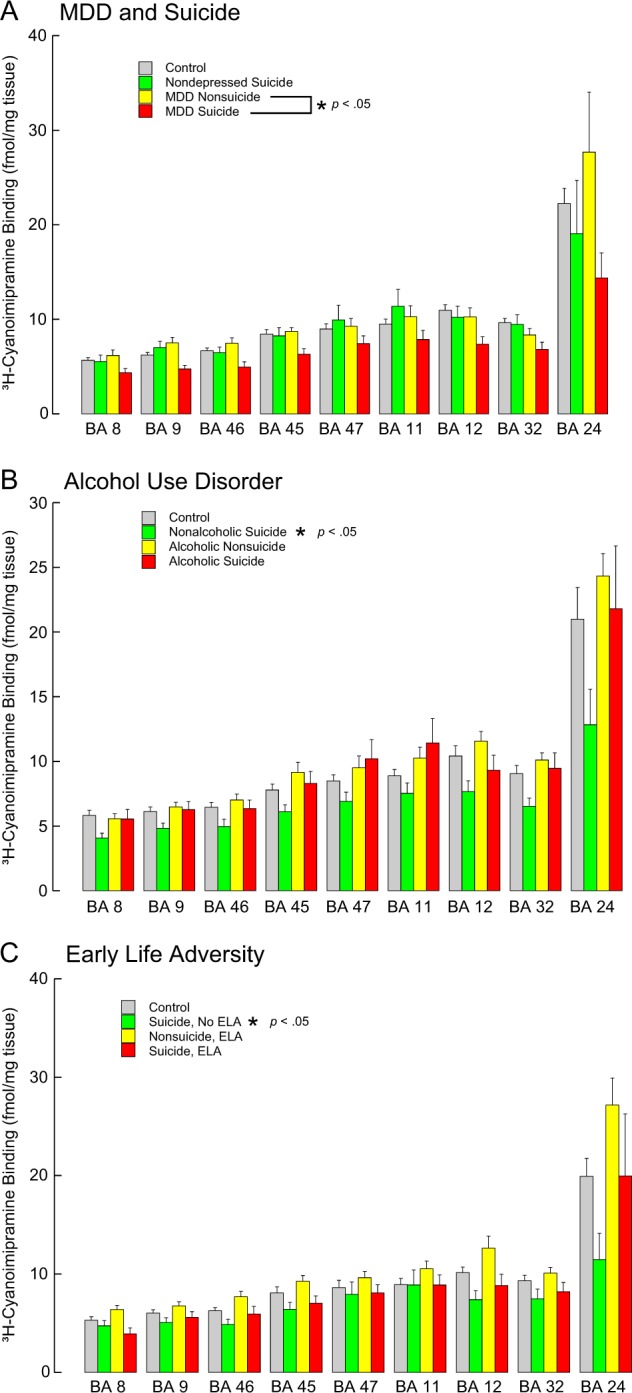
Serotonin transporter (SERT) binding in the prefrontal cortex. **A** suicide and major depressive disorder (MDD), **B** alcohol use disorder and **C** early life adversity (ELA). SERT sites were labeled with [^3^H]-Cyanoimipramine. Note that: SERT is less in depressed suicide decedents and with MDD. With AUD there is more SERT but only in suicides, and there is more in ELA but only in nonsuicides. Values are expressed as mean ± SEM

Including all cases, and adjusting for sex and age, SERT binding was lower in suicides (*F* = 15.367, df = 1,9, *p* = 0.004). There was no interaction between suicide and Brodmann area (*F* = 0.828, df = 8,1665, *p* = 0.578) indicating the effect of suicide was comparable in all areas. Limiting the cases to only those with psychological autopsy, the effect of suicide remained significant (*F* = 11.464, df = 1,9, *p* = 0.008, Fig. 2a).

#### Major depressive disorder

There was lower SERT binding with MDD (*F* = 9.476, df = 1,13, *p* = 0.009), and the lower binding was found in all brain regions (region by depression interaction *F* = 0.378, df = 8,8, *p* = 0.878). In the model with suicide and MDD, the effect of suicide was not significant (*F* = 5.147, df = 1,9, *p* = 0.050) suggesting that lower SERT in suicide is attributable, at least in part, to the MDD diagnosis (Table 3, Fig. 2a).

#### Alcohol use disorder

There were cases with AUD in both nonsuicides (*n* = 53 with AUD) and suicides (*n* = 20 with AUD). The AUD diagnosis was associated with more SERT (Fig. 2b, Table 4; F = 8.135, df = 1,16, *p* = 0.012); the AUD interaction with brain region was not significant (F = 0.671, df = 8,10, *p* = 0.708) indicating the effect of AUD was comparable in all areas. In the same model, the suicide effect adjusted for AUD was not significant (F = 2.972, df = 1,10, *p* = 0.117), suggesting the AUD effect is in nonsuicides (Fig. 2b). Similarly, with MDD in the model, the diagnosis of MDD was also no longer significant (F = 3.418, df = 1,23, *p* = 0.078). This may reflect the lower rate of MDD in the alcoholics in our sample. In nonalcoholics, 34 of 40 suicides had MDD. In alcoholics, 14 of 58 had MDD.

**Table 4:** Transporter, 5-HT_1A_ and 5-HT_2A_ binding in suicide and major depressive disorder in subjects with and without alcohol use disorder

Only alcoholic cases:											
Receptor	n	Group	BA 8	BA 9	BA 46	BA 45	BA 47	BA 11	BA 12	BA 32	BA 24
SERT	52	NonSuicide	5.56 ± 2.01	6.48 ± 2.59	7.02 ± 2.95	9.14 ± 5.55	9.51 ± 6.36	10.24 ± 5.87	11.56 ± 4.48	10.1 ± 3.89	24.32 ± 8.93
	20	Suicide	5.54 ± 2.57	6.27 ± 2.75	6.36 ± 2.57	8.29 ± 4.06	10.2 ± 6.65	11.42 ± 8.29	9.3 ± 4.99	9.46 ± 5.37	21.81 ± 16.08
5-HT_1A_	49	NonSuicide	13.04 ± 4.43	14.6 ± 4.65	13.65 ± 3.79	14.59 ± 4.39	17.07 ± 5.51	18.9 ± 5.97	17.19 ± 5.37	15.29 ± 5.41	18.81 ± 7.44
	20	Suicide	15.96 ± 3.38	16.15 ± 3.89	16.31 ± 3.34	15.6 ± 2.8	19.72 ± 4.46	22.18 ± 6.24	18.77 ± 4.96	17.53 ± 3.85	22.24 ± 4.73
5-HT_2A_	52	NonSuicide	26.31 ± 10.13	28.46 ± 11.36	29.77 ± 9.91	28.65 ± 9.76	32.01 ± 10.6	33.14 ± 10.84	33.43 ± 10.21	28.74 ± 9.93	21.84 ± 8.28
	19	Suicide	38.94 ± 12.19	36.59 ± 12.73	38.32 ± 12.07	35.53 ± 10.59	39.52 ± 11.59	38.9 ± 10.53	38.84 ± 12	34.99 ± 11.33	30.2 ± 10.99
SERT	57	NoMDD	5.62 ± 2.08	6.49 ± 2.65	6.99 ± 2.88	9.18 ± 5.41	9.83 ± 6.59	10.75 ± 6.49	11.52 ± 4.73	10.39 ± 3.92	23.97 ± 10.57
	14	MDD	5.35 ± 2.66	6.13 ± 2.68	6.12 ± 2.87	7.44 ± 3.88	9.12 ± 6.09	9.85 ± 7.55	8.13 ± 3.97	7.97 ± 5.54	19.91 ± 12.92
5-HT_1A_	55	NoMDD	13.33 ± 4.16	14.7 ± 4.43	14.15 ± 3.91	14.74 ± 4.17	17.42 ± 4.96	19.47 ± 5.63	17.41 ± 5.22	15.42 ± 4.98	18.97 ± 6.78
	13	MDD	16.66 ± 4.52	16.53 ± 4.72	15.57 ± 3.61	15.65 ± 3.3	19.67 ± 6.71	21.51 ± 8.49	18.83 ± 5.74	18.24 ± 5.22	24.03 ± 6.03
5-HT_2A_	56	NoMDD	27.93 ± 12.35	29.78 ± 12.48	31.22 ± 11.23	29.54 ± 10.53	33.49 ± 11.81	34.32 ± 11.57	34.56 ± 11.52	29.74 ± 10.81	23.05 ± 9.42
	14	MDD	36.21 ± 9.18	33.82 ± 11.3	35.97 ± 11.11	34.53 ± 9.52	36.68 ± 9.77	36.66 ± 8.93	36.97 ± 8.89	32.3 ± 10.01	26.92 ± 9.42

Only non-alcoholic cases											
Receptor	n	Group	BA 8	BA 9	BA 46	BA 45	BA 47	BA 11	BA 12	BA 32	BA 24
SERT	50	NonSuicide	5.82 ± 2.19	6.11 ± 2.59	6.45 ± 2.5	7.79 ± 3.17	8.49 ± 3.28	8.89 ± 3.38	10.41 ± 5.29	9.06 ± 4.42	21 ± 13.42
	36	Suicide	4.07 ± 1.73	4.82 ± 2.42	4.97 ± 3.19	6.1 ± 3.2	6.9 ± 4.05	7.53 ± 4.67	7.66 ± 4.64	6.52 ± 3.51	12.83 ± 10.65
5-HT_1A_	51	NonSuicide	12.6 ± 3.16	13.44 ± 3.97	12.94 ± 3.9	13.57 ± 4.16	15.94 ± 5.11	17.11 ± 5.67	17.64 ± 6.48	14.37 ± 4.68	17.6 ± 5.59
	37	Suicide	13.83 ± 4.22	14.19 ± 4.49	15.09 ± 4.72	15.03 ± 3.89	17.41 ± 4.85	18.84 ± 5.14	18.74 ± 4.82	15.27 ± 4.1	17.7 ± 5.98
5-HT_2A_	52	NonSuicide	28.25 ± 8.14	28.38 ± 9.39	28.61 ± 9.93	30.68 ± 10.47	32.2 ± 10.65	32.84 ± 11.83	32.55 ± 12.12	29.97 ± 8.76	26.72 ± 8.6
	38	Suicide	26.92 ± 12.7	29.14 ± 13.43	30.39 ± 13	29.57 ± 12.48	32.96 ± 13.38	33.34 ± 13.83	31.88 ± 13.46	29.16 ± 13.21	18.85 ± 6.4
SERT	55	NoMDD	5.68 ± 2.17	6.16 ± 2.6	6.33 ± 2.53	7.64 ± 3.15	8.36 ± 3.31	8.81 ± 3.41	10.29 ± 5.15	8.86 ± 4.39	20.02 ± 13.28
	31	MDD	4.04 ± 1.82	4.57 ± 2.26	4.93 ± 3.3	6.15 ± 3.32	6.97 ± 4.14	7.48 ± 4.8	7.48 ± 4.82	6.52 ± 3.54	13.49 ± 11.53
5-HT_1A_	55	NoMDD	12.71 ± 3.14	13.58 ± 3.97	13.22 ± 3.94	13.87 ± 4.01	16.37 ± 5.1	17.58 ± 5.46	17.91 ± 6.38	14.66 ± 4.6	17.53 ± 5.42
	33	MDD	13.81 ± 4.48	14.05 ± 4.58	14.87 ± 4.91	14.69 ± 4.27	16.86 ± 4.96	18.21 ± 5.62	18.39 ± 4.91	14.86 ± 4.24	17.91 ± 6.43
5-HT_2A_	56	NoMDD	28.1 ± 8.47	28.26 ± 9.63	28.42 ± 10.03	30.41 ± 10.36	32.06 ± 10.68	32.76 ± 11.83	32.14 ± 12.13	29.51 ± 8.82	26.36 ± 8.56
	34	MDD	27.03 ± 12.85	29.44 ± 13.54	30.93 ± 13.16	29.9 ± 12.92	33.27 ± 13.57	33.54 ± 14.02	32.52 ± 13.6	29.89 ± 13.53	17.7 ± 5.63

Values are expressed as fmol/mg tissue, mean ± SD

*BA* Brodmann area, *MDD* major depressive disorder, *SERT* serotonin transporter

#### Adversity

When childhood adversity was included in the model along with suicide, there were no SERT differences related to childhood adversity (*F* = 1.907, df = 1,9, *p* = 0.20, Table 5). The suicide effect of less SERT binding was significant (*p* = 0.006) and there was a suicide*adversity interaction (*p* = 0.007) with adversity associated with more SERT binding, but only in non-suicides (Fig. 2c). The interaction term with Brodmann area was significant with the difference localized to BA24.

**Table 5:** Transporter, 5-HT_1A_ and 5-HT_2A_ binding in suicide and early life adversity

Receptor	*n*	Group	BA 8	BA 9	BA 46	BA 45	BA 47	BA 11	BA 12	BA 32	BA 24
SERT	67	Nonsuicide, No ELA	5.3 ± 2.17	6.03 ± 2.67	6.26 ± 2.53	8.07 ± 4.99	8.63 ± 5.68	8.93 ± 4.85	10.15 ± 4.04	9.3 ± 4.63	19.94 ± 10.87
	31	Suicide, No ELA	4.73 ± 2.33	5.06 ± 2.7	4.85 ± 2.76	6.4 ± 3.7	7.93 ± 6.55	8.88 ± 7.97	7.39 ± 4.67	7.48 ± 5.19	11.45 ± 10.04
	31	Nonsuicide, ELA	6.38 ± 1.87	6.76 ± 2.28	7.69 ± 2.85	9.26 ± 3.2	9.63 ± 3.45	10.52 ± 4.26	12.62 ± 6.15	10.1 ± 3.2	27.17 ± 11.65
	19	Suicide, ELA	3.91 ± 1.71	5.59 ± 2.54	5.93 ± 3.27	7.03 ± 3.21	8.07 ± 3.66	8.88 ± 4.51	8.81 ± 4.76^a^	8.19 ± 3.86	19.96 ± 16.71
5-HT_1A_	67	Nonsuicide, No ELA	12.68 ± 3.81	13.47 ± 4.15	12.69 ± 3.77	13.47 ± 4.09	15.3 ± 4.87	16.48 ± 5.24	16.28 ± 5.96	14.08 ± 4.84	17.3 ± 6.62
	31	Suicide, No ELA	14.5 ± 3.85	14.7 ± 4.98	14.81 ± 5.06	15.01 ± 3.97	18.52 ± 5.12	20.59 ± 6.44	19.04 ± 5.23	15.93 ± 4.76	19.69 ± 6.11
	30	Nonsuicide, ELA	12.64 ± 3.58	14.98 ± 4.64	14.45 ± 3.83	15.22 ± 4.51	18.33 ± 4.68	20.36 ± 5.3	19.51 ± 5.21	16.14 ± 5.19	19.1 ± 5.45
	20	Suicide, ELA	14.18 ± 2.95	14.86 ± 3.79	15.94 ± 3.02	15.19 ± 3.08	17.46 ± 4.38	19.04 ± 4.92	17.88 ± 4.25	15.64 ± 2.71	18.21 ± 5.83
5-HT_2A_	69	Nonsuicide, No ELA	27.74 ± 8.84	28.15 ± 10.11	28.49 ± 9.71	29.72 ± 10.34	31.79 ± 10.76	32.94 ± 11.31	32.43 ± 11.01	29.39 ± 8.77	25.74 ± 8.63
	31	Suicide, No ELA	25.51 ± 11.68	27.07 ± 12.35	28.65 ± 12.75	27.07 ± 11.59	30.7 ± 12.58	31.16 ± 13.51	29.08 ± 11.61	26.08 ± 11.73	19.49 ± 8.54
	31	Nonsuicide, ELA	27.47 ± 9.92	29.5 ± 11.05	30.58 ± 10.54	30.19 ± 10.15	33.77 ± 10.01	34.03 ± 11.3	33.81 ± 11.94	30.13 ± 10.45	22.37 ± 8.89
	20	Suicide, ELA	37.18 ± 12.78	37.89 ± 12.89	37.01 ± 12.5	37.05 ± 10.66	41.2 ± 12.07	40.73 ± 10.35	39.34 ± 12.73	35.76 ± 11.89	29.21 ± 10.45

Values are expressed as fmol/mg tissue, mean ± SD

*BA* Brodmann area, *ELA* early live adversity, *SERT* serotonin transporter

#### Sex, age, aggression

Both sex and age were significant in every statistical model examined. Females (*n* = 49) had less SERT binding than males (*n* = 171, *b* =−0.33, t = −4.27, df = 216, *p* < 0.0001) in all BA areas, except BA24 (post hoc test, *p* = 0.069). SERT binding declined with age (t = −3.04, df = 215, *p* = 0.0027), and the effect was uniform across brain regions (age by BA interaction *p* > 0.05). SERT positively correlated with aggression in BA9 (*r* = 0.193, *p* = 0.0220), BA46 (*r* = 0.270, *p* = 0.0020), BA45 (*r* = 0.179, *p* = 0.0350), BA11 (r = 0.173, *p* = 0.0460) and BA32 (*r* = 0.198, *p* = 0.022) in cases overall.

### 5-HT_1A_ receptors

In nonsuicides (*n* = 143), 5-HT_1A_ binding ranged from 13.9 ± 4.8 fmol/mg tissue in BA8 to 19.5 ± 7.2 fmol/mg tissue in BA24 (Table 3).

#### Suicide

**Fig. 3:**
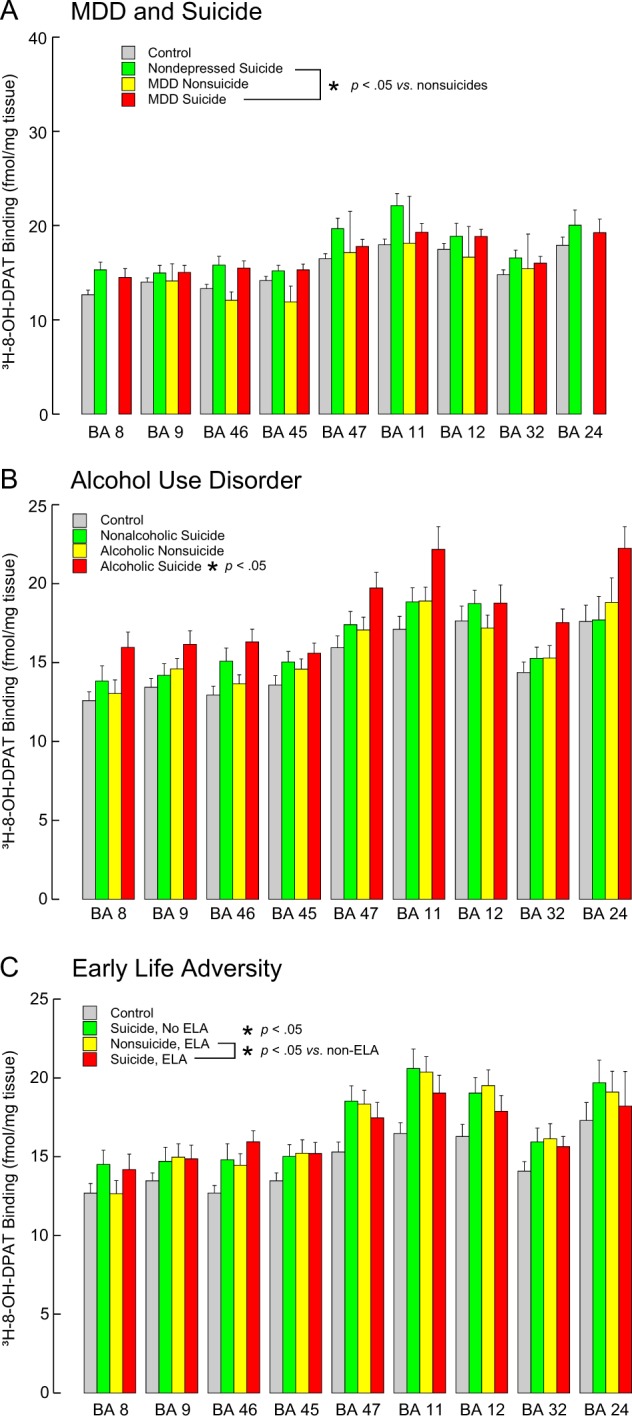
5-HT_1A_ receptor binding in the prefrontal cortex. **A** suicide and major depressive disorder (MDD), **B** alcohol use disorder and **C** early life adversity (ELA). 5-HT_1A_ receptors were labeled with [^3^H]-8-OH-DPAT. There is no data for BA8 or BA24 for MDD nonsuicides because there was only one case with these regions. Note: 5-HT_1A_ binding was greater in suicides independent of MDD, AUD was associated with higher 5-HT_1A_ binding but only in suicides, and ELA is associated with more 5-HT_1A_ receptor binding. Values are expressed as mean ± SEM

There was no significant effect of suicide (*F* = 0.163, df = 1,10, *p* = 0.695) and the suicide by brain region interaction term was not significant (*F* = 0.270, df = 8,1714, *p* = 0.976). However, in the sensitivity analysis limited to cases with psychological autopsy data, suicide was significant with more 5-HT_1A_ binding (*F* = 60.049, df = 1,14, *p* < 0.001) and the suicide by brain region interaction was not significant (*F* = 0.320, df = 8,1179, *p* = 0.959, Fig. 3a).

#### Major depressive disorder

5-HT_1A_ binding was not different in MDD (F = 2.148, df = 1,12, *p* = 0.168) and there was no significant interaction between suicide and MDD (F = 2.680, df = 1,12, *p* = 0.128) or interaction between MDD and Brodmann area (F = 0.909, df = 8,8, *p* = 0.552, Table 3, Fig. 3a).

#### Alcohol use disorder

With AUD in the model with suicide, MDD and sex, AUD was associated with more 5-HT_1A_ binding (*F* = 35.703, df = 1,20, *p* < 0.001) and the interaction with brain region was not significant (*F* = 1.852, df = 8,2, *p* = 0.397, Fig. 3b, Table 4). The effect of suicide was still significant (*F* = 18.054, df = 1,12, *p* = 0.001) and there was a significant interaction between suicide and AUD (*F* = 16.562, df = 1,19, *p* < 0.001) with AUD associated with more 5-HT_1A_ binding but only in suicides. There was no effect of MDD (*p* = 0.696) and there was no interaction between suicide and MDD (*p* = 0.087), but there was an interaction between MDD and AUD (*F* = 53.894, df = 1,18, *p* < 0.001) with more 5-HT_1A_ binding only in MDD cases with AUD.

#### Adversity

With adversity in the model, suicide was associated with higher 5-HT_1A_ binding (*F* = 27.697, df = 1,14, *p* < 0.001), adversity was not significant (*F* = 0.619, df = 1,28, *p* = 0.438, Table 5). There was no interaction between suicide and Brodmann area (*F* = 0.319, df = 8,8, *p* = 0.937). There was an interaction between suicide and adversity (*F* = 14.133, df = 1,9, *p* = 0.004) with adversity associated with more 5-HT_1A_ binding, but only in non-suicides (Fig. 3c). There was no interaction between adversity and brain region (*F* = 0.273, df = 8,8, *p* = 0.958).

#### Sex, age, aggression

Sex was significant in select models particularly those examining the effect of suicide and AUD. 5-HT_1A_ binding was negatively correlated with age in BA9, BA46 and BA32 (Pearson correlation values −0.141, −0.147, −0.151, *p* values 0.037, 0.037 and 0.029 respectively). 5-HT_1A_ binding correlated positively with lifetime aggression in BA9 (*r* = .299, *p* < 0.001), BA46 *r* = .259, *p* = 0.003), BA45 (*r* = .194, *p* = 0.023), BA47 (*r* = .258, *p* = 0.002), BA11 (r = .212, *p* = 0.014), BA24 (*r* = .248, *p* = 0.036) and BA32 (*r* = .179, *p* = 0.036).

### 5-HT_2A_ receptors

#### Suicide

**Fig. 4:**
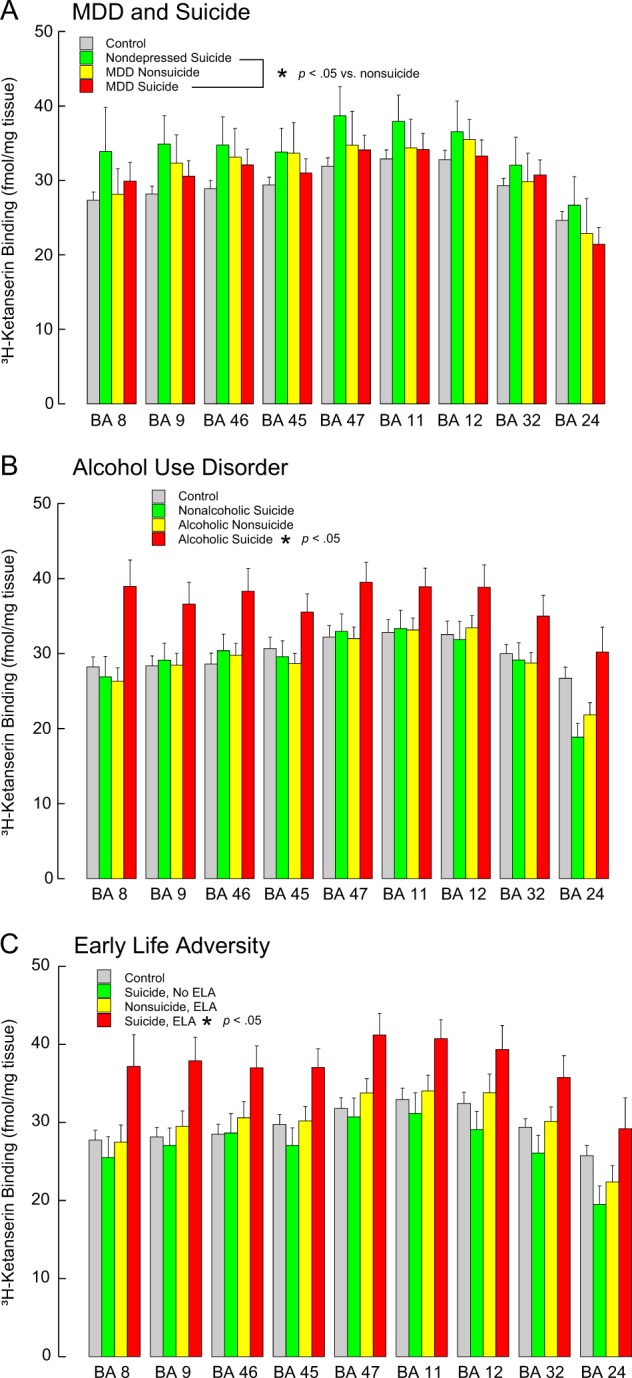
5-HT_2A_ receptor binding in the prefrontal cortex. **A** suicide and major depressive disorder (MDD), **B** alcohol use disorder and **C** early life adversity (ELA). 5-HT_2A_ receptors were labeled with [^3^H]-Ketanserin. Note that: there is more 5-HT_2A_ binding in suicides but no difference with MDD; there is more binding in AUD and ELA, but only in suicides. Values are expressed as mean ± SEM

There was no suicide effect detected overall (*F* = 2.139, df = 1,9, *p* = 0.176) and no suicide by region interaction (*F* = 0.477, df = 8,1687, *p* = 0.873). However, in the subgroup with psychological autopsies, there was more 5-HT_2A_ binding in suicides (*F* = 41.226, df = 1,12, *p* < 0.001, Table 3). There was no suicide by region interaction (*F* = 0.386, df = 8,1193, *p* = 0.929) suggesting the greater amount of binding in suicide was widespread and the diagnosis of cases was important (Fig. 4a).

#### Major depressive disorder

With MDD in the model, higher 5-HT_2A_ binding was still significant with suicide (*F* = 18.695, df = 1,30, *p* < 0.001) and there was no suicide:Brodmann area interaction (*F* = 0.510, df = 8,8, *p* = 0.820). There was no significant effect of MDD (*F* = 2.570, df = 1,39, *p* = 0.117) and no MDD by brain region interaction (*F* = 0.361, df = 8,8, *p* = 0.914). This suggests that the diagnosis of MDD does not explain higher 5-HT_2A_ binding in suicides (Fig. 4a).

#### Alcohol use disorder

There was no significant association between AUD and 5-HT_2A_ binding (*F* = 2.302, df = 1,17, *p* = 0.148, Table 4); there was no interaction between AUD and Brodmann area on binding (*F* = 0.206, df = 8,1193, *p* = 0.990). With AUD in the model with suicide and MDD, the effect of suicide remained and MDD became significant (*F* = 17.59, df = 1,313, *p* < 0.001), and there was an interaction between AUD and suicide (*F* = 31.427, df = 1,130, *p* < 0.001) with more 5-HT_2A_ binding in suicides with AUD (Fig. 4b), but not in suicides without AUD. There was no interaction between AUD and MDD (*F* = 0.687, df = 1,33, *p* = 0.413).

#### Adversity

Adversity was associated with more 5-HT_2A_ binding (*F* = 25.484, df = 1,78, *p* < 0.001); the brain region by adversity interaction term was not significant (*F* = 0.743, df = 8,8, *p* = 0.658, Table 5). With adversity and suicide in the model, higher 5-HT_2A_ binding remained associated with suicide (*F* = 37.658, df = 1,12, *p* < 0.001). There was an interaction between suicide and childhood adversity (*F* = 201.938, df = 1,21, *p* < 0.001) such that higher 5-HT_2A_ binding was in suicides with adversity regardless of Brodmann area examined (Fig. 4c).

#### Sex, age, aggression

5-HT_2A_ receptor binding negatively correlated with age in all regions (*r* = −0.227 to −0.441, *p* = 2.43 × 10^−9^ to 7.9 × 10^−12^). Females (*n* = 48) had lower 5-HT_2A_ binding than males (*n* = 175) (*b* = −0.14, *t* = −2.32, df = 218, *p* = 0.0210). 5-HT_2A_ binding correlated positively with aggression in all brain regions (*r* = 0.270–0.396, *p* < 0.05) except BA24 (*r* = 0.199, *p* = 0.085).

## Discussion

The main findings in the present study were: (1) SERT binding was lower in suicides independent of sex, but dependent on MDD diagnosis; higher SERT binding is associated with AUD; (2) 5-HT_1A_ binding was greater in suicides, independent of MDD, while AUD was associated with higher 5-HT_1A_ binding but only in suicides; (3) 5-HT_2A_ binding was greater in suicides only when accounting for the effects of MDD and AUD in the model. (4) Reported childhood adversity was associated with higher SERT, 5-HT_1A_ binding, and 5-HT_2A_ binding. These findings illustrate that the ability to detect differences in SERT, 5-HT_1A_, and 5-HT_2A_ binding in the brain between cases with mood disorders and dying by suicide is dependent on Axis I diagnosis and reported childhood adversity, therein demonstrating the importance of clinical characterization of both cases and controls under investigation. Different findings in alcoholism from depression and suicide indicate distinct serotonin system pathophysiology.

### Serotonin transporter

We previously found lower SERT binding in suicides in ventral PFC^26,27,29^. We confirm and extend this, finding the lower SERT is widespread and appears associated more with MDD than with suicide. Lower SERT in suicides is reported by some investigators, but not all (see^12,36,37^ for review). Our current study suggests one factor contributing to the discrepancy between studies is the proportion of MDD cases in the suicide group. Imaging studies in nonfatal attempters also report less SERT^22,38–40^. Interestingly, Miller et al.^20^ reported less SERT in depressed suicide attempters but not depressed nonattempters compared to controls, raising the possibility that SERT is related more to suicidal behavior than to depression. We observed a *p*-value of 0.053 which did not reach statistical significance but arguably is suggestive of an effect of suicide. Our findings here regarding suicide and MDD suggest that low SERT throughout the PFC is related to MDD more than to suicide, and the suicide effect may be the result of a more pronounced difference in the ventral PFC. Hypofunction of the ventral PFC may lead to increased suicide risk due to the inability to restrain the self-destructive act.

In AUD, we found more SERT binding, but others report lower SERT in cerebral cortex^41^. No difference in alcoholics or alcoholic-suicides in SERT mRNA was found in BA9 or BA24^42^, but there was a negative association between SERT mRNA in BA24 and anxiety symptoms. In in vivo imaging studies, alcoholics had less [11C]McN5652 binding to the SERT^43^. Others^44,45^ did not find any difference in [11C]DASB binding to the SERT in alcoholics. Comorbidity with mood disorders may contribute to the inconsistent findings and future studies of AUD should consider effects of comorbid MDD since the two diagnoses may have opposite effects on binding.

### 5-HT_1A_ receptors

We observed more 5-HT_1A_ binding in suicides, but only in cases that underwent psychological autopsy. We did not detect a difference in 5-HT_1A_ binding in MDD, but we found more 5-HT_1A_ binding associated with AUD. Several investigators, though not all, report higher 5-HT_1A_ binding in depressed suicides (for review see ref. ^36^). We previously reported^26,27^ higher ^3^H-8-OH-DPAT binding in suicide that was anatomically restricted to ventrolateral prefrontal cortex, as were the increases reported by others^46,47^. Parsey et al.^48^ found that MDD patients whose depression did not remit had higher 5-HT_1A_ binding and also an over-representation of GG genotype, suggesting that genotype may also affect the level of receptor binding, symptom progression and treatment response. Negative reports did not examine binding in anatomically discrete areas or did not examine ventral prefrontal cortex^49–52^. The lack of agreement about elevated 5-HT_1A_ binding raises the possibility that the differences associated with suicide are anatomically discrete. The radioligand used may also affect receptor binding since antagonist but not agonist binding is decreased in MDD, suggesting binding differences may reflect the G-protein coupled or uncoupled state of the receptor^53^. Lower 5-HT_1A_ receptor binding is reported in the cerebral cortex in alcoholics^4,49,54^. Lastly, we did not find differences in 5-HT_1A_ binding when all cases and controls were examined together, greater 5-HT_1A_ binding in suicides was only found when restricting the analysis to only those cases diagnosed by psychological autopsy. We believe this demonstrates how cases without psychological autopsy can bias, obscure or otherwise influence the outcome of an analysis to the point of affecting the conclusion reached.

In vivo PET imaging studies report higher 5-HT_1A_ binding in depressed MDD subjects and remitted MDD;^55^ others find less binding^56–58^. The different results may be due to the method of estimating binding^55^. We observed more 5-HT_1A_ binding associated with childhood adversity in nonsuicides, and a rodent study of stress in infants finds increased gene expression^59^.

We find more 5-HT_1A_ binding with AUD, as did Thompson et al.^42^ who found higher 5-HT_1A_ mRNA in BA9 in AUD but not AUD-suicides and not in BA24. Martinez and colleagues did not find any differences in 5-HT_1A_ density in humans with alcohol dependence^45^. Taken together, the findings suggest brain region is important as is the potential for the presence of alcohol status to obscure effects of suicide. Genomic studies may be helpful for understanding 5HT_1A_ regulation in suicide, MDD and AUD.

### 5-HT_2A_ receptors

We found higher 5-HT_2A_ binding in suicide, but only in cases with psychological autopsy. Other studies report higher^28,60,61^, lower^62^ and no difference^52^ in 5-HT_2A_ binding in suicide. Both higher and lower binding are also reported in MDD in vivo (see ref. ^15^ for review). We detected more 5-HT_2A_ binding in AUD in the present study but not previously^31^. No difference in 5-HT_2A_ binding was reported in AUD by others^63^. Most studies did not separate effects of depression or alcoholism from suicide. Therefore, we extend previous observations by finding that 5-HT_2A_ binding is greater in suicide alcoholics and in suicides with ELA but is not increased with MDD. Another possible explanation for the discrepancies is the use of an agonist *versus* antagonist ligand; higher binding in suicide was detected using the agonist LSD^28,64^, while the antagonist Ketanserin detected lower or no change in suicide. Another possible explanation for the discrepancy is the ligand specificity. It is known that ketanserin has affinity for tetrabenazine receptors, alpha_1_ adrenergic receptors and histamine receptors. We blocked for these receptors during the incubations. Some of the findings in the literature, including our own, were obtained by incubation of tissue using ^125^I-LSD^28^. A problem with ^125^I-labeled ligands is that differences in tissue thickness result in a darker image and higher measured receptor density. While this may pose less of a problem for small pieces of tissue, when sectioning entire hemispheres, inevitably there will be parts of the tissue that are thicker producing a darker image. We found a positive correlation between 5-HT_2A_ binding and aggression raising the possibility that increased 5-HT_2A_ binding in suicide is associated with the increased aggression commonly associated with suicide behavior^30^. Alternatively, the discrepant findings may be due to childhood adversity exposure. This not only implies a relationship between the 5-HT_2A_ receptor and brain development and/or the development of AUD, but also demonstrates the importance of the diagnostic composition of the sample under study.

### Early life adversity (ELA)

We found that reported ELA was associated with more SERT, 5-HT_1A_ and 5-HT_2A_ receptor binding. Reported ELA was associated with lower SERT in vivo in MDD^65^. Childhood adversity was not associated with 5-HT_1A_ receptor density^16^, and early maternal separation, an animal model of childhood adversity in humans, was not associated with difference in 5-HT_1A_ mRNA gene expression in rats^66^. In peer-reared rhesus monkeys, another animal model of childhood adversity, 5-HT_1A_ receptor binding was less in father-reared compared with mother-reared animals, but greater in peer-reared females^67^.

Gene-environment interactions are increasingly found between early life stress and risk for psychiatric illness, including MDD (see ref. ^68^ for review) and suicide risk (see ref. ^69^). An interaction is widely reported between lower expressing alleles of the SERT gene promotor variant (5-HTTLPR), stressful early life events and increased risk for MDD^70,71^ and suicide^72^. Interestingly, it has been found that the S allele and the early adversity effects can be additive suggesting persistent changes in SERT expression may relate to altered serotonergic neurotransmission levels which could bring about increased disease risk^73^. No gene-environment interaction is reported between the 5-HT_1A_^74^ or 5-HT_2A_^75^ receptor gene polymorphisms and MDD and childhood stress. Alternatively, innate receptor binding level may mitigate the effects of gene x environment interactions.

### Sex

We found females have less SERT and 5-HT_2A_ binding than males. In contrast, sex was not a significant determinant in most of the models in which we examined the 5-HT_1A_ receptor. Males and females differ in their prevalence in psychiatric illnesses (see refs. ^68,76–78^) and in serotonergic receptor densities^79,80^. We previously reported sex differences in SERT and 5-HT_1A_^26^. In vivo receptor binding studies as measured by PET, report that women have more 5-HT_1A_ receptors^81^ and fewer SERT^80^ and 5-HT_2A_ receptors^82^ compared with men. Others do not find sex differences^49,83,84^.

### Conclusions

Reduced serotonergic neurotransmission has been a long-standing hypothesis in the etiology of suicide and mood disorders. The SERT is located on axons and axon terminals and are an indication of serotonergic innervation and intrasynaptic serotonin levels^85^. Less SERT in depressed suicides therefore suggests less innervation or greater SERT internalization secondary to less intra-synaptic 5-HT. 5-HT_1A_ and 5-HT_2A_ receptors in the PFC are located predominantly on cortical interneurons. We previously reported more 5-HT_1A_ and 5-HT_2A_ receptor binding in the PFC of depressed suicides, but only when we corrected for the density of cortical interneurons^86^. 5-HT_1A_ receptor activation results in hyperpolarization and a decrease in neuronal activity in PFC^87^ on pyramidal neurons and cortical interneurons.

The causes of receptor differences in suicide is unclear. Evidence for receptor up- and down- regulation comes from several sources. Serotonin receptor down regulation has long been suggested as a mechanism of action for antidepressant drugs (see refs. ^88–90^). By the converse, in classic theories of receptor regulation, reduced 5-HT agonism or long term receptor blockade may lead to up-regulation of postsynaptic receptors, but the mechanisms of receptor regulation are complicated, differ by receptor and even by brain region for the same receptor and are not addressed in the current study (for reviews see refs. ^90–94^).

An increase in 5-HT_1A_ receptors in PFC suggests an inhibition of excitatory output from cortical regions that mediate executive function and behavioral restraint. We hypothesize that reduced cortical activity may be a top-down cause of a reduction in restraint and increase in the risk for suicide behavior. Cognitive testing has been used to find that suicide attempters have worse inhibitory control in executive functioning skills;^95^ see ref. ^96^ for review). Neuroimaging studies of brain structure report prefrontal deficits in suicide behavior including impaired decision making and social assessment^97,98^. We now report altered 5-HT receptors in the same prefrontal cortex regions. Multimodal imaging studies of structure and connectivity in suicide attempters^1^, such as those used in connectomic studies^99,100^, support this model.

### Strengths and limitations

In the present study we could compare effects across brain regions related to MDD, suicide, AUD, and childhood adversity and consider the impact of age and sex because we were able to draw upon the largest known collection of human postmortem cases with quantitative receptor autoradiography and psychological autopsy data. Our findings demonstrate the importance of clinical characterization of all cases, including the “controls”. We and others have shown how lack of reproducible findings in postmortem studies of suicide can be attributed to effects of antemortem factors, PMI, toxicology, neuropathology, clinical diagnosis, brain region identification and even freezer storage time^13,101–103^. The larger group size studied here, not only provided more statistical power for detecting differences, it accounted for potential receptor binding effects associated with comorbid diagnoses by using psychological autopsy to identify the presence of any comorbid illnesses and then including the diagnosis in the statistical model of the outcome measure. Effects detected in the sensitivity analysis, that were not detected in the analysis of the larger sample, which included cases that had not undergone psychological autopsy, further illustrates that undetected diagnoses can introduce bias in results that can obscure detection of differences between groups. The inclusion of alcoholics and subjects with other comorbid diagnoses provided the opportunity to begin to understand how additional illness burden can change the associated brain receptors, but a more clinically homogeneous sample increased the detectability of receptor differences. Regardless of the reliability and validity of the psychological autopsy procedure, it is not infallible, and the possibility remains that there are cases, for example suffering from an MDD episode at the time of death, that were not diagnosed or detected whether or not the psychological autopsy was performed, and these types of cases could affect the outcome. Postmortem studies have the inherent limitation of being cross-sectional and cannot address cause and effect relationships. Another limitation is not knowing the cellular sources giving rise to the receptors being measured as is coming through laser capture microdissection and cellular fractionation. Receptor binding is an endpoint measure and does nothing to address whether differences reflect changes in transcription, translation or posttranslational modification. Likewise, receptor expression is under genetic and epigenetic control and none of this is examined here, nor can it be.

Future work should examine SERT, 5HT_1A_, and 5HT_2A_ binding in nonfatal suicide attempts in mood disorders and other diagnoses, and consider the effects of reported childhood adversity to determine the extent that any differences are part of these diagnostic entities or the diathesis for suicidal behavior. Indeed, there are several reports in the literature examining clinical similarities and differences between suicide attempters and completers^104–106^, and neurochemical similarities and differences^107–109^. The presence of differences in attempters to completers will suggest the differences are not only present antemortem, but will also be informative as to whether there is a continuum of differences in the brain in suicide and completers represent the most extreme form of the behavior. Future potential therapeutic approaches to the prevention of suicide, the treatment of MDD or AUD and reversing effects of childhood adversity, could then be developed to target these molecules.

## Supplementary material

Supplementary Figure Legends

Figure S1

Figure S2

Supplementary Information
