# Supplementary material for: Serotonin receptors and suicide, major depression, alcohol use disorder and reported early life adversity
Source: Transl Psychiatry. 2018 Dec 14;8:279. doi: 10.1038/s41398-018-0309-1 (PMC6294796; doi:10.1038/s41398-018-0309-1)
Supplement: Supplementary file 1 — Supplementary Figure Legends [file 41398_2018_309_MOESM1_ESM.docx]

**Supplementary Figures**

**Figure S1**. Schematic illustration of the effects of suicide and major depressive disorder diagnosis (MDD), on the serotonin transporter (SERT), 5-HT1A and 5-HT2A receptor in prefrontal cortex. The upper left diagram depicts a coronal section through a hemisphere and identifies the Brodmann areas present at this anatomical level. Note that suicide is associated with no difference in the SERT, and more 5-HT1A and 5-HT2A binding throughout the prefrontal cortex, but only in analyses including cases and controls undergoing psychological autopsy. MDD is associated with less SERT throughout the prefrontal cortex.

**Figure S2**. Schematic illustration of the effects of alcohol use disorder (AUD) and early life adversity (ELA) on the serotonin transporter (SERT), 5-HT1A and 5-HT2A receptor in prefrontal cortex. Note that diagnosis of AUD is associated with widespread elevated SERT, 5-HT1A and 5-HT2A receptor binding throughout the prefrontal cortex. ELA is associated with more SERT in nonsuicides, more 5-HT1A receptor binding and more 5-HT2A receptor binding.
