# Supplementary material for: Serotonin receptors and suicide, major depression, alcohol use disorder and reported early life adversity
Source: Transl Psychiatry. 2018 Dec 14;8:279. doi: 10.1038/s41398-018-0309-1 (PMC6294796; doi:10.1038/s41398-018-0309-1)
Supplement: Supplementary file 2 — Figure S1 [file 41398_2018_309_MOESM2_ESM.pdf]

Figure S1

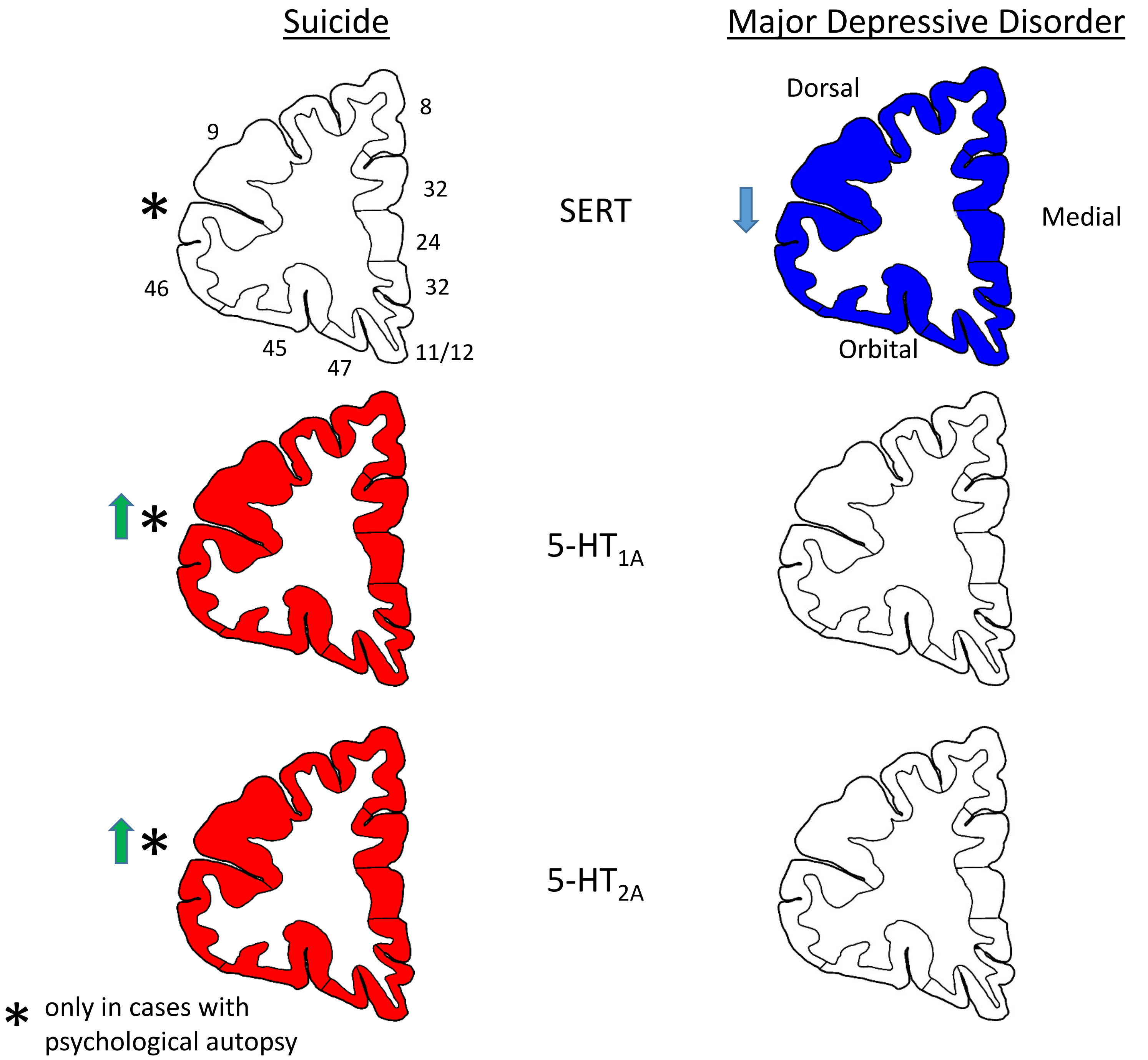
Suicide
Major Depressive Disorder
9
8
32
24
32
46
45
47
11/12
*
Dorsal
Medial
Orbital
SERT
5-HT1A
5-HT2A
*
*
* only in cases with psychological autopsy
