# Supplementary material for: Serotonin receptors and suicide, major depression, alcohol use disorder and reported early life adversity
Source: Transl Psychiatry. 2018 Dec 14;8:279. doi: 10.1038/s41398-018-0309-1 (PMC6294796; doi:10.1038/s41398-018-0309-1)
Supplement: Supplementary file 3 — Figure S2 [file 41398_2018_309_MOESM3_ESM.pdf]

Figure S2

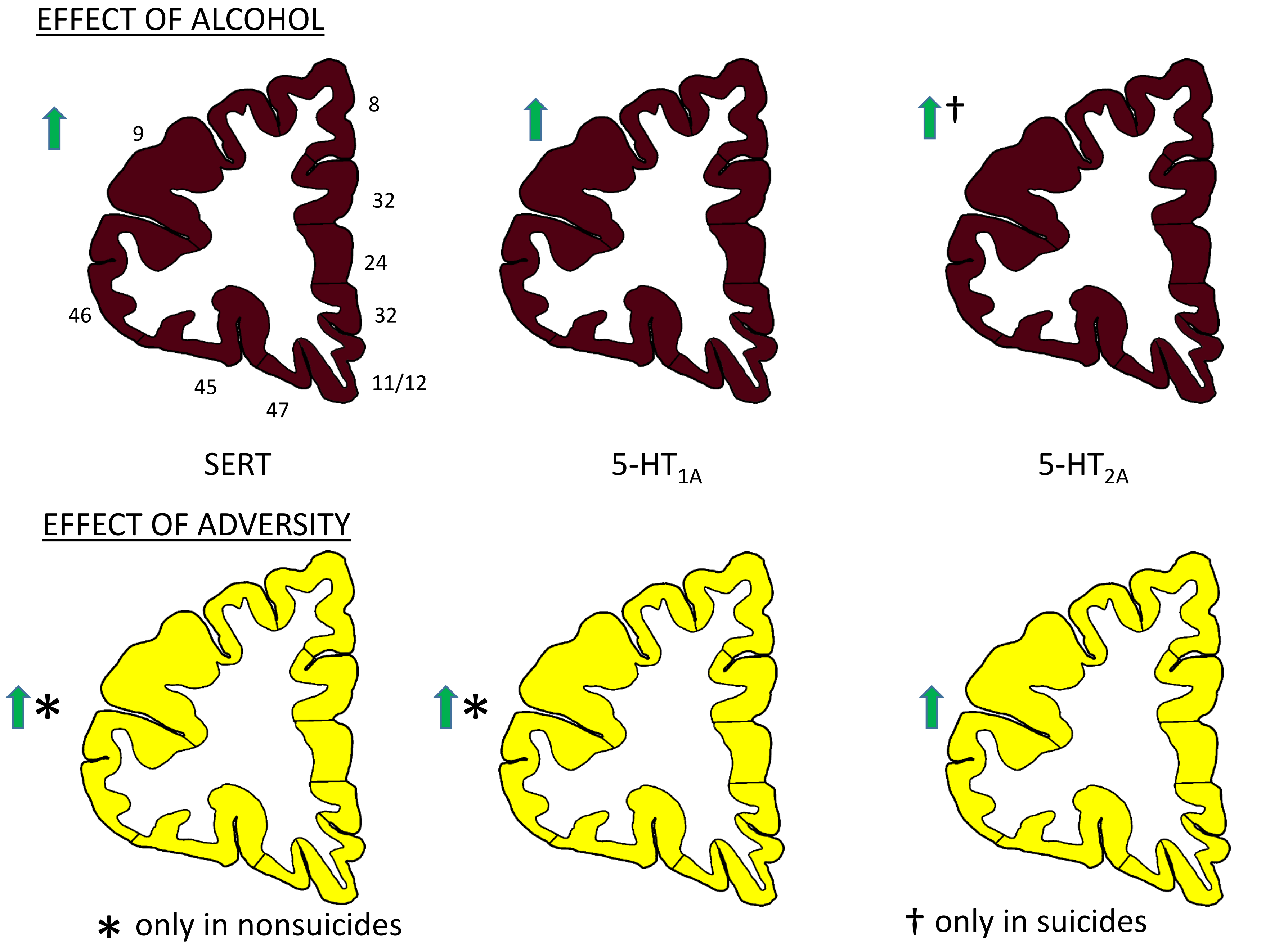
EFFECT OF ALCOHOL
9
8
32
24
46
32
45
47
11/12
†
SERT
5-HT1A
5-HT2A
EFFECT OF ADVERSITY
*
*
* only in nonsuicides
† only in suicides
