# Supplementary material for: Serotonin receptors and suicide, major depression, alcohol use disorder and reported early life adversity
Source: Transl Psychiatry. 2018 Dec 14;8:279. doi: 10.1038/s41398-018-0309-1 (PMC6294796; doi:10.1038/s41398-018-0309-1)
Supplement: Supplementary file 4 — Supplementary Information [file 41398_2018_309_MOESM4_ESM.docx]

|  | **SERT** | | **5-HT_1A_** | | **5-HT_2A_** | |
| --- | --- | --- | --- | --- | --- | --- |
| **All Cases** |  |  |  |  |  |  |
| Age | F_1,1665_ = 8.285 | ***p* = .004** | F_1,1714_ = 14.670 | ***p* < .0001** | F_1,1687_ = 290.288 | ***p* < .0001** |
| Sex | F_1,1665_ = 41.148 | ***p* < .0001** | F_1,1714_ = 38.414 | ***p* < .0001** | F_1,1687_ = 8.929 | ***p* = 0.003** |
| Suicide | F_1,8.834_ = 15.367 | ***p* = .004** | F_1,10.356_ = .163 | *p* = .695 | F_1,9.351_ = 2.139 | *p* = 0.176 |
| Brodmann Area | F_8,8.004_ = 112.936 | ***p* < .0001** | F_1,8.008_ = 100.340 | ***p* < .0001** | F_8,8.01_ = 16.672 | ***p* < .0001** |
| Suicide * Brodmann Area | F_8,1665_ = 0.828 | *p* = .578 | F_8,1714_ = .270 | *p* = .976 | F_8,1687_ = 0.477 | *p* = 0.873 |
|  |  |  |  |  |  |  |
| **Psychological Autopsy Cases** |  |  |  |  |  |  |
| Age | F_1,1175_ = 18.005 | ***p* < .0001** | F_1,1179_ = 91.215 | ***p* < .0001** | F_1,1193_ = 229.799 | ***p* < .0001** |
| Sex | F_1,1175_ = 20.466 | ***p* < .0001** | F_1,1179_ = 6.029 | ***p*** = **.014** | F_1,1193_ = 4.618 | ***p* = 0.032** |
| Suicide | F_1,8.834_ = 11.464 | ***p* = .008** | F_1,14.448_ = 60.049 | ***p* < .0001** | F_1,12.325_ = 41.226 | ***p* < .0001** |
| Brodmann Area | F_8,8.004_ = 27.240 | ***p* < .0001** | F_8,8.028_ = 70.877 | ***p* < .0001** | F_8,8.017_ = 16.048 | ***p* < .0001** |
| Suicide * Brodmann Area | F_8,1175_ = 2.129 | ***p*** = **.031** | F_8,1179_ = .320 | *p* = .959 | F_8,1193_ = 0.386 | *p* = 0.929 |
|  |  |  |  |  |  |  |
| **Psychological Autopsy Cases, with MDD** | |  |  |  |  |  |
| Age | F_1,1148_ = 15.705 | ***p* < .0001** | F_1,1152_ = 90.027 | ***p* < .0001** | F_1,1166_ = 223.527 | ***p* < .0001** |
| Sex | F_1,1148_ = 15.263 | ***p* < .0001** | F_1,1152_ = 6.576 | ***p*** = **.010** | F_1,1166_ = 4.005 | *p* = 0.046 |
| Suicide | F_1,8.724_ = 5.147 | ***p* = .050** | F_1,12.259_ = .490 | *p* = .497 | F_1,29.772_ = 18.695 | ***p* < .0001** |
| Depressed | F_1,13.297_ = 9.476 | ***p* = .009** | F_1,12.209_ = 2.148 | *p* = .168 | F_1,39.332_ = 2.57 | *p* = 0.117 |
| Brodmann Area | F_8,4.873_ = 16.405 | ***p* = .004** | F_8.2.161_ = 8.124 | *p* = .101 | --- |  |
| Suicide * Depressed | F_1,10.567_ = 10.449 | ***p* = .008** | F_1.11.896_ = 2.680 | *p* = .128 | F_1,20.393_ = 0.066 | *p* = 0.800 |
| Suicide * Brodmann Area | F_8,7.998_ = 3.414 | ***p* = .05** | F_8.8.001_ = .949 | *p* = .528 | F_8,7.988_ = 0.51 | *p* = 0.820 |
| Depressed * Brodmann Area | F_8,7.999_ = .378 | *p = .905* | F_8.8.000_ = .909 | *p* = .552 | F_8,7.998_ = 0.361 | *p* = 0.914 |
| Suicide * Depressed * Brodmann Area | F_8,1148_ = .593 | *p =* .785 | F_8,1152_ = 1.506 | *p* = .151 | F_8,1166_ = 0.245 | *p* = 0.982 |

**Statistics Outcomes on Primary Measures**

|  |  | **SERT** | | **5-HT_1A_** | | **5-HT_2A_** | |
| --- | --- | --- | --- | --- | --- | --- | --- |
| **Psych Autopsy Cases, Alcoholism** | | |  |  |  |  |  |
| Age | | F_1,1175_ = 11.479 | ***p* = .001** | F_1,1179_ = 80.438 | ***p* < .0001** | F_1,1193_ = 222.127 | ***p* < .0001** |
| Sex | | F_1,1175_ = 27.085 | ***p* < .0001** | F_1,1179_ = 14.938 | ***p* < .0001** | F_1,1193_ = 1.768 | *p* = 0.184 |
| Alcoholic | | F_1,8.709_ = 13.951 | ***p* = .005** | F_1,9.759_ = 8.219 | ***p* = 0.017** | F_1,16.766_ = 2.302 | *p* = 0.148 |
| Brodmann Area | | F_8,8.005_ = 10.713 | ***p* < .0001** | F_8,8.015_ = 26.851 | ***p* < .0001** | F_8,8.058_ = 32.02 | ***p* < .0001** |
| Alcoholic * Brodmann Area | | F_8,1175_ = 1.862 | *p* = .062 | F_8,1179_ = 0.898 | *p* = 0.517 | F_8,1193_ = 0.206 | *p* = 0.990 |
|  | |  |  |  |  |  |  |
| **Psych Autopsy Cases, Suicide, MDD, Alcoholism** | | | | |  |  |  |
| Age | | F_1,1114_ = 11.598 | ***p* = .001** | F_1,1118_ = 94.553 | ***p* < .0001** | F_1,1132_ = 220.677 | ***p* < .0001** |
| Sex | | F_1,1114_ = 15.888 | ***p* < .0001** | F_1,1118_ = 8.19 | ***p* = .004** | F_1,1132_ = 2.859 | *p* = 0.091 |
| Suicide | | F_1,9.587_ = 2.972 | *p* = .117 | F_1,12.138_ = 18.054 | ***p* = .001** | F_1,43.984_ = 13.444 | ***p* = 0.001** |
| Depressed | | F_1,22.578_ = 3.418 | *p* = .078 | F_1,11.27_ = 0.696 | *p* = .421 | F_1,313.437_ = 17.59 | ***p* < .0001** |
| Alcoholic | | F_1,15.843_ = 8.135 | ***p* = .012** | F_1,19.588_ = 35.703 | ***p* < .0001** | F_1,46.377_ = 6.65 | ***p* = 0.013** |
| Brodmann Area | | F_8,1.595_ = 23.294 | *p* = .069 | F_8,5.704_ = 4.487 | ***p* = .046** | **---** |  |
| Suicide * Depressed | | F_1,13.194_ = 1.905 | *p* = .190 | F_1,12.509_ = 3.458 | *p* = .087 | F_1,21.02_ = 0.996 | *p* = 0.330 |
| Suicide * Alcoholic | | F_1,13.007_ = 1.193 | *p* = .295 | F_1,19.175_ = 16.562 | ***p* = .001** | F_1,130.469_ = 31.427 | ***p* < .0001** |
| Suicide * Brodmann Area | | F_8,13.740_ = 1.397 | *p* = .280 | F_8,8.079_ = 1.011 | *p* = .494 | F_8,12.796_ = 0.298 | *p* = 0.953 |
| Depressed * Alcoholic | | F_1,12.620_ = 1.639 | *p* = .224 | F_1,18.313_ = 53.894 | ***p* < .0001** | F_1,33.362_ = 0.687 | *p* = 0.413 |
| Depressed * Brodmann Area | | F_8,11.695_ = .229 | *p* = .977 | F_8,6.257_ = 1.394 | *p* = .348 | F_8,10.177_ = 0.072 | *p* = 0.999 |
| Alcoholic * Brodmann Area | | F_8,10.022_ = .671 | *p* = .708 | F_8,2.005_ = 1.852 | *p* = .397 | F_8,0.425_ = 5.044 | *p* = 0.541 |
| Suicide * Depressed * Alcoholic | | F_1,6.717_ = 8.721 | ***p* = .022** | F_1,6.46_ = 32.983 | ***p* = .001** | F_1,7.813_ = 4.367 | *p* = 0.071 |
| Suicide * Depressed * Brodmann Area | | F_8,3.267_ = 4.444 | *p* = .111 | F_8,9.705_ = 1.978 | *p* = .157 | F_8,2.726_ = 4.844 | *p* = 0.126 |
| Suicide * Alcoholic * Brodmann Area | | F_8,0.826_ = 11.457 | *p* = .274 | F_8,2.153_ = 2.051 | *p* = .356 | --- |  |
| Depressed * Alcoholic * Brodmann Area | | F_8,3.688_ = 4.560 | *p* = .090 | F_8,4.059_ = 1.27 | *p* = .434 | F_8,2.001_ = 2.846 | *p* = 0.286 |
| Suicide * Depressed * Alcoholic * Brodmann Area | | F_6,1114_ = .177 | *p* = .983 | F_6,1118_ = 0.305 | *p* = .934 | F_6,1132_ = 0.089 | *p* = 0.997 |

|  | **SERT** | | **5-HT_1A_** | | **5-HT_2A_** | |
| --- | --- | --- | --- | --- | --- | --- |
| **Psychological Autopsy Cases, Suicide, Adversity** | | |  |  |  |  |
| Age | F_1,1077_ = 17.691 | ***p* < .0001** | F_1,1090_ = 79.694 | ***p* < .0001** | F_1,1100_ = 166.883 | ***p* < .0001** |
| Sex | F_1,1077_ = 14.651 | ***p* < .0001** | F_1,1090_ = 2.475 | *p* = 0.116 | F_1,1100_ = 4.874 | ***p* = 0.027** |
| Suicide | F_1,8.531_ = 13.133 | ***p* = .006** | F_1,14.37_ = 27.697 | ***p* < .0001** | F_1,12.395_ = 37.658 | ***p* < .0001** |
| Adversity | F_1,9.136_ = 1.907 | *p* = .200 | F_1,27.61_ = 0.619 | *p* = 0.438 | F_1,78.33_ = 25.484 | ***p* < .0001** |
| Brodmann Area | F_8,15.160_ = 7.319 | ***p* < .0001** | **---** |  | F_8,5.404_ = 19.223 | ***p* = 0.002** |
| Suicide * Adversity | F_1,21.247_ = 8.826 | ***p* = .007** | F_1,9.482_ = 14.133 | ***p* = 0.004** | F_1,20.812_ = 201.938 | ***p* < .0001** |
| Suicide * Brodmann Area | F_8,8.018_ = 19.369 | ***p* < .0001** | F_8,8.005_ = 0.319 | *p* = 0.937 | F_8,8.025_ = 2.695 | *p* = 0.091 |
| Adversity * Brodmann Area | F_8,8.030_ = 21.878 | ***p* < .0001** | F_8,8.003_ = 0.273 | *p* = 0.958 | F_8,8.042_ = 0.743 | *p* = 0.658 |
| Suicide * Adversity * Brodmann Area | F_8,1077_ = .161 | *p* = .996 | F_8,1090_ = 0.964 | *p* = 0.463 | F_8,1100_ = 0.131 | *p* = 0.998 |
|  |  |  |  |  |  |  |
| **Psychological Autopsy Cases, Adversity** | |  |  |  |  |  |
| Age | F_1,1095_ = 16.302 | ***p* < .0001** | F_1,1108_ = 80.062 | ***p* < .0001** | F_1,1118_ = 161.161 | ***p* < .0001** |
| Suicide | F_1,1095_ = 22.062 | ***p* < .0001** | F_1,1108_ = 9.886 | ***p* = 0.002** | F_1,1118_ = 6.09 | ***p* = 0.014** |
| Adversity | F_1,8.974_ = 2.149 | *p* = .177 | F_1,16.251_ = 1.385 | *p* = 0.256 | F_1,20.999_ = 1.704 | *p* = 0.206 |
| Brodmann Area | F_8,8.001_ = 17.568 | ***p* < .0001** | F_8,8.021_ = 40.111 | ***p* < .0001** | F_8,8.023_ = 21.044 | ***p* < .0001** |
| Adversity * Brodmann Area | F_8,1095_ = 3.609 | ***p* < .0001** | F_8,1108_ = 0.52 | *p* = 0.842 | F_8,1118_ = 0.314 | *p* = 0.961 |

|  | **SERT** | | **5-HT_1A_** | | **5-HT_2A_** | |
| --- | --- | --- | --- | --- | --- | --- |
| **Psychological Autopsy Cases, Suicide, MDD, Adversity** |  | |  |  |  |  |
| Age | F_1,1043_ = 13.234 | ***p* < .0001** | F_1,1058_ = 77.326 | ***p* < .0001** | F_1,1066_ = 154.175 | ***p* < .0001** |
| Sex | F_1,1043_ = 11.078 | ***p* = 0.001** | F_1,1058_ = 2.559 | *p* = 0.110 | F_1,1066_ = 6.34 | ***p* = 0.012** |
| Suicide | F_1,8.2_ = 3.516 | *p* = 0.097 | F_1,21.229_ = 36.058 | ***p* < .0001** | F_1,6.593_ = 13.573 | ***p* = 0.009** |
| Depressed | F_1,7.556_ = 37.439 | ***p* < .0001** | F_1,15.064_ = 15.718 | ***p* = 0.001** | F_1,0.11_ = 375.302 | *p* = 0.594 |
| Adversity | F_1,10.234_ = 0.017 | *p* = 0.897 | F_1,58.325_ = 0.034 | *p* = 0.854 | F_1,173.487_ = 2.645 | *p* = 0.106 |
| Brodmann Area | F_8,0.395_ = 21.437 | *p* = 0.422 | F_8,0.345_ = 27.119 | *p* = 0.442 | --- |  |
| Suicide * Depressed | F_1,8.043_ = 3.303 | *p* = 0.106 | F_1,7.907_ = 23.004 | ***p* = 0.001** | F_1,7.757_ = 12.095 | ***p* = 0.009** |
| Suicide * Adversity | F_1,10.802_ = 0.437 | *p* = 0.522 | F_1,14.993_ = 6.712 | ***p* = 0.020** | F_1,43.058_ = 87.298 | ***p* < .0001** |
| Suicide * Brodmann Area | F_8,15.047_ = 0.434 | *p* = 0.883 | F_8,2.303_ = 0.916 | *p* = 0.610 | F_8,15.447_ = 0.179 | *p* = 0.991 |
| Depressed * Adversity | F_1,10.189_ = 2.202 | *p* = 0.168 | F_1,15.339_ = 0.097 | *p* = 0.760 | F_1,24.719_ = 38.77 | ***p* < .0001** |
| Depressed * Brodmann Area | F_8,14.238_ = 0.144 | *p* = 0.995 | F_8,1.461_ = 1.756 | *p* = 0.464 | F_8,10.325_ = 0.072 | *p* = 1.000 |
| Adversity * Brodmann Area | F_8,13.454_ = 1.231 | *p* = 0.353 | F_8,3.275_ = 0.440 | *p* = 0.845 | F_8,5.782_ = 0.415 | *p* = 0.875 |
| Suicide * Depressed * Adversity | F_1,7.019_ = 36.561 | ***p* = 0.001** | F_1,6.22_ = 0.022 | *p* = 0.887 | F_1,7.001_ = 5.605 | ***p* = 0.05** |
| Suicide * Depressed * Brodmann Area | F_8,42.251_ = 5.473 | ***p* < .0001** | F_6,6_ = 0.45 | *p* = 0.823 | F_8,37.585_ = 1.209 | *p* = 0.321 |
| Suicide * Adversity * Brodmann Area | F_8,0.152_ = 100.966 | *p* = 0.596 | F_8,3.699_ = 2.133 | *p* = 0.254 | --- |  |
| Depressed * Adversity * Brodmann Area | F_8,5.441_ = 16.926 | ***p* = 0.002** | F_8,5.481_ = 1.398 | *p* = 0.361 | F_8,1.118_ = 6.474 | *p* = 0.268 |
| Suicide * Depressed * Adversity * Brodmann Area | F_6,1043_ = 0.079 | *p* = 0.998 | F_6,1058_ = 0.321 | *p* = 0.926 | F_6,1066_ = 0.075 | *p* = 0.998 |

--- SPSS could not compute the error degrees of freedom

* interaction

Abbreviations: MDD: Major Depressive Disorder; SERT: serotonin transporter

| **Pearson Correlation Coefficients (PCC)** | | | | | | |  | | | | | |  | | | | | |
| --- | --- | --- | --- | --- | --- | --- | --- | --- | --- | --- | --- | --- | --- | --- | --- | --- | --- | --- |
|  | **SERT** | | | | | | **5-HT_1A_** | | | | | | **5-HT_1A_** | | | | | |
|  | **Age** | | | **Aggression** | | | **Age** | | | **Aggression** | | | **Age** | | | **Aggression** | | |
|  | **PCC** | **Sig. (2-tailed)** | **n** | **PCC** | **Sig. (2-tailed)** | **n** | **PCC** | **Sig. (2-tailed)** | **n** | **PCC** | **Sig. (2-tailed)** | **n** | **PCC** | **Sig. (2-tailed)** | **n** | **PCC** | **Sig. (2-tailed)** | **n** |
| BA 8 | **-0.0274**** | **0.001** | **140** | 0.039 | 0.732 | 80 | -0.073 | 0.385 | 142 | 0.219 | 0.053 | 79 | **-.391**** | **0.000** | **154** | **.396**** | **0.000** | **92** |
| BA 9 | **-0.0259**** | **0.000** | **216** | **0.193*** | **0.022** | **142** | **-0.141*** | **0.037** | **220** | **0.299**** | **0.000** | **143** | **-.441**** | **0.000** | **219** | **.337**** | **0.000** | **144** |
| BA 46 | **-0.209**** | **0.004** | **186** | **0.270**** | **0.002** | **128** | **-0.147*** | **0.037** | **202** | **0.259**** | **0.003** | **129** | **-.422**** | **0.000** | **184** | **.339**** | **0.000** | **126** |
| BA 45 | -0.092 | 0.184 | 211 | **0.179*** | **0.035** | **140** | -0.13 | 0.059 | 212 | **0.194*** | **0.023** | **136** | **-.389**** | **0.000** | **210** | **.271**** | **0.001** | **137** |
| BA 47 | -0.044 | 0.525 | 210 | **0.187*** | **0.03** | **136** | -0.06 | 0.388 | 210 | **0.258**** | **0.002** | **136** | **-.422**** | **0.000** | **207** | **.312**** | **0.000** | **131** |
| BA 11 | -0.052 | 0.459 | 206 | **0.173*** | **0.046** | **134** | -0.01 | 0.881 | 209 | **0.212*** | **0.014** | **133** | **-.409**** | **0.000** | **205** | **.270**** | **0.002** | **132** |
| BA 12 | -0.077 | 0.303 | 183 | 0.149 | 0.107 | 118 | 0.047 | 0.519 | 194 | 0.133 | 0.133 | 129 | **-.364**** | **0.000** | **193** | **.289**** | **0.001** | **126** |
| BA 32 | -0.11 | 0.115 | 207 | **0.198*** | **0.022** | **135** | **-0.151*** | **0.029** | **209** | **0.179*** | **0.036** | **137** | **-.384**** | **0.000** | **211** | **.286**** | **0.001** | **136** |
| BA 24 | -0.087 | 0.334 | 125 | 0.195 | 0.101 | 72 | 0.019 | 0.828 | 131 | **0.248*** | **0.036** | **72** | **-.227**** | **0.010** | **128** | 0.199 | 0.085 | 76 |
| * Correlation is significant at the 0.05 level (2-tailed). | | | | | |  |  |  |  |  |  |  |  |  |  |  |  |  |
| ** Correlation is significant at the 0.01 level (2-tailed). | | | | | |  |  |  |  |  |  |  |  |  |  |  |  |  |
